# Local and Systemic Oxidative Stress Biomarkers for Male Infertility: The ORION Study

**DOI:** 10.3390/antiox11061045

**Published:** 2022-05-25

**Authors:** Anna T. Bergsma, Hui Ting Li, Jitske Eliveld, Marian L. C. Bulthuis, Annemieke Hoek, Harry van Goor, Arno R. Bourgonje, Astrid E. P. Cantineau

**Affiliations:** 1 Department of Obstetrics and Gynecology, University of Groningen, University Medical Centre Groningen, 9700 RB Groningen, The Netherlands; a.t.bergsma@umcg.nl (A.T.B.); h.t.li@umcg.nl (H.T.L.); j.eliveld@umcg.nl (J.E.); a.hoek@umcg.nl (A.H.); 2 Department of Pathology and Medical Biology, University of Groningen, University Medical Center Groningen, 9713 GZ Groningen, The Netherlands; m.bulthuis01@umcg.nl (M.L.C.B.); h.van.goor@umcg.nl (H.v.G.); 3 Department of Gastroenterology and Hepatology, University of Groningen, University Medical Center Groningen, 9713 GZ Groningen, The Netherlands; a.r.bourgonje@umcg.nl

**Keywords:** male infertility, oxidative stress, free thiols, malondialdehyde, reactive oxygen species

## Abstract

Infertility problems occur in around 10% of all couples worldwide, with male-factor infertility as the sole contributor in 20–30% of these cases. Oxidative stress (OS) is suggested to be associated with the pathophysiology of male infertility. In spermatozoa, OS can lead to damage to the cell membrane, resulting in disruption of DNA integrity and a decrease in motility. Established biomarkers for OS include free thiols and malondialdehyde (MDA), both representing different components of the reactive species interactome (RSI). This exploratory study aimed to investigate seminal plasma-free thiol and MDA levels in relation to semen parameters as defined by the World Health Organization (WHO) to determine if these markers are adequate to define local OS status. Furthermore, this study investigated if there is a relation between systemic and local OS status by comparing seminal concentrations of free thiol (R-SH, sulfhydryl groups, representing the extracellular redox status) and MDA (lipid peroxidation product) levels to those measured in serum. Free thiol and MDA measurements in both serum and semen plasma were performed in 50 males (18–55 y) of couples seeking fertility treatment. A significant positive correlation was found between seminal plasma-free thiol levels and sperm concentration and progressive motility (r = 0.383, *p* = 0.008 and r = 0.333, *p* = 0.022, respectively). In addition, a significant positive correlation was found between MDA levels in seminal plasma and sperm concentration (r = 0.314, *p* = 0.031). This study supports that seminal plasma-free thiols may be promising as local OS biomarkers. No associations were observed between local and systemic OS biomarker concentrations.

## 1. Introduction

Infertility is defined as the inability to conceive after one year of unprotected intercourse [1]. It occurs in around 10% of all couples worldwide. Male-factor infertility is the sole contributor to 20–30% of all infertility cases [2]. Regardless of the known etiologic factors causing male infertility, such as genetic and chromosomal disorders, testis descent disorders, and post-infection factors, approximately 50% of the cases of male infertility are categorized as idiopathic [3]. Oxidative stress (OS) is a process associated with the etiology of male infertility. In spermatozoa, oxidative stress can lead to concurrent damage to proteins and lipids of the cell membrane, causing a reduction in plasma membrane fluidity and an increase in permeability. Reduced membrane fluidity results in a decrease in motility and ability to fuse with the oocyte, whereas increased membrane permeability results in a disruption of the cells’ DNA integrity [4,5].

When discussing the role of OS in the pathophysiology of male infertility, it is important to distinguish between systemic and local OS. Systemic OS is caused by various lifestyle-related factors, such as smoking and excessive alcohol use, and environmental factors such as exposure to toxic substances, such as the heavy metals cadmium and lead [4]. Smoking is, in turn, associated with reduced sperm count and an increase in the number of morphological defects of spermatozoa [6]. However, the direct relation between systemic OS and semen quality is still unclear. Examples of causes for enhanced local production of oxidative stressors, e.g., reactive oxygen species (ROS), are systemic as well as local infections in the male genital tract and smoking leading to the attraction of excess leukocytes and increased abnormal spermatozoa with excessive mitochondrial production of ROS present in semen plasma [7,8,9,10,11]. ROS also damage the blood-testis barrier (BTB), resulting in infiltration, directly affecting spermatogenesis [12].

The extent of OS is commonly measured by two biomarkers representing different components of the ‘reactive species interactome’ (RSI): free thiols, representing potent scavengers of ROS; considered reflective of the whole-body redox status and malondialdehyde (MDA), a damage marker of OS representing an end-product of lipid peroxidation [7,10]. Free thiols (R-SH, sulfhydryl groups) and MDA are redox-related biomarkers present in both blood serum and seminal plasma. Free thiols in blood serum have proven utility as a biomarker for systemic OS in various OS-related conditions [13,14,15]. Furthermore, free thiols do not only serve as potent ROS scavengers, but they are also believed to act as central hubs of inter-organ redox communication, serving as multimodal redox relays by kinetically controlling intra- and extracellular redox exchange reactions. They transduce a variety of redox-regulated events mainly via oxidative protein modifications, covering both short-term (e.g., alterations of protein structure and activity) and longer-term (e.g., regulation of gene expression) biological adaptations, thereby representing a global read-out of the whole-body redox status [7]. Seminal plasma-free thiol levels are positively correlated with sperm concentration, morphology, and progressive motility [16,17,18,19]. Putatively, reduced systemic free thiols may also reflect the local redox status in seminal plasma. Moreover, systemic MDA is increased in blood serum in states of increased OS (such as hypoxia) and is negatively correlated with sperm motility and positively correlated with morphologic abnormalities and apoptosis [20,21,22,23]. MDA is a well-established biomarker of lipid peroxidation, representing an end-product of ROS-inflicted damage to polyunsaturated fatty acids in cells. MDA is the prototype marker of thiobarbituric acid reactive substances (TBARS) and is highly correlated with other biomarkers of lipid peroxidation such as 4-hydroxy-nonenal (HNE) and F_2_-isoprostanes (e.g., 15(*S*)-8-*iso*-prostaglandin-F_2α_) [21].

To date, no study has investigated the relation between systemic and local OS in semen plasma as well as the association between both biomarkers. Therefore, it is unclear whether the oxidant-antioxidant mechanisms systemically and locally in the male genital tract are correlated or whether these systems rather individually regulate their oxidative stress state. An association between systemic and local oxidative stress status is important to relate general health and lifestyle factors to semen quality (e.g., sperm concentration, motility, and morphology). Subsequently, it provides insight into the significance of free thiol and MDA levels regarding semen quality in male infertility or as potential monitoring tools for therapeutic modulation and lifestyle interventions, such as smoking cessation, on semen quality.

The first aim of this exploratory study was to investigate seminal plasma-free thiol and MDA levels in relation to semen parameters of males seeking fertility treatment and to determine whether free thiol and MDA levels are adequate biomarkers to define local oxidative stress status [24]. Subsequently, we aimed to investigate systemic OS status in relation to the local OS status, as reflected by measured concentrations of free thiol and MDA levels in blood serum and seminal plasma, respectively.

## 2. Materials and Methods

### 2.1. Study Population

From December 2020 until August 2021, 50 males from couples seeking fertility treatment at the Centre of Reproductive Medicine (CRM) of the University Medical Center Groningen (UMCG) were invited to participate in the study. Eligible participants were between 18 and 55 years old, with a planned semen analysis in the context of standard infertility diagnostic workup. Males who receive(d) chemo- and/or radiotherapy, use(d) testosterone supplementation and/or anabolic steroids, and an abnormal semen analysis due to genetic causes were not eligible for this study.

This study received ethical approval from the Institutional Review Board (IRB) of the University Medical Center Groningen (UMCG) (in Dutch: “Medisch Ethische Toetsingscommissie”, METc, IRB approval no. 2020/568). All participants provided written informed consent for their participation in the study. The study was performed in accordance with the principles of the Declaration of Helsinki (2013).

### 2.2. Study Parameters and Laboratory Procedures

The main study parameters were free thiol concentrations and MDA concentrations in serum and seminal plasma. Semen analysis (SA) parameters, measured according to the WHO guidelines (2021), were used to assess sperm quality [24,25]. Seminal plasma was collected after 15 min of centrifugation at 1625× *g* (Hettich Zentrifuge universal 32 centrifugation machine).

The blood samples had to coagulate an hour before processing. Serum was collected after centrifugation for 10 min at 1885× *g*. The serum and semen samples were stored at a temperature of −80 °C within two hours after blood and semen collection. A pseudonymized list was created to ensure all serum and semen samples were traceable to a specific participant.

Free thiol concentrations in both blood serum and seminal plasma were measured using the modified Ellman technique as previously described by Ellman (1959) and Hu et al. (1993) but with minor modifications [13,26,27]. First, serum samples were diluted at 1:4 with 0.1 M pH 8.2 Tris buffer. Seminal plasma samples were not diluted. Subsequently, 90 μL of both samples were added to a 96-well microtiter plate. The background absorption was measured at 412 nm with a reference measurement at 630 nm. After this, 20 µL 1.9 mM 5,5′-dithio-bis (2-nitrobenzoic acid) (DTNB, Ellman’s reagent, CAS-number 69-78-2, Sigma Aldrich Corporation, St. Loui, MO, USA) in phosphate buffer (0.1 M, pH 7.0) was added. Next, the samples were incubated for 20 min at room temperature, after which the absorption was measured again. Free thiol concentrations were determined by parallel measurement of an L-cysteine standard solution (CAS-number 52-90-4, Fluka Biochemika, Buchs, Switzerland) calibration curve (using a concentration range from 15.625 to 1000 µM) in 0.1 M Tris, 10 mM EDTA (pH 8.2). Blood serum-free thiol concentrations were corrected for albumin concentrations, which were measured according to standard procedures, and presented as µmol/g of albumin. This correction was performed because circulating proteins largely influence the total concentration of potentially detectable free thiols, and albumin is the most abundant protein present in blood [28]. Seminal plasma-free thiol concentrations were not corrected for albumin concentration since albumin concentrations in seminal plasma are substantially lower than in blood serum. Seminal plasma-free thiol levels were expressed as µmol/L or μM.

The MDA levels in both blood serum and seminal plasma were measured using the thiobarbituric acid reactive substance (TBARS) assay previously described by Janero (1990), using the assay kit from Cayman Chemical Company (#700870) [29]. The standard reagents were prepared for colorimetric assay. In total, 250 µL of the thiobarbituric acid (TBA) MDA standard was diluted with 750 µL of water to create the stock solution of 125 µM. For the assay, 100 µL of the sample (serum or semen plasma) or standard was added to 1.5 mL microcentrifuge vials. Thereafter, 100 µL of trichloroacetic acid (TCA) Assay Reagent (10%) was added to each vial, followed by 800 µL of the Color Reagent. After this, the vials were vortexed and put in boiled water for one hour. Next, the vials were placed in an ice bath to incubate for 10 min to stop the reaction. Subsequently, the vials were centrifuged for 10 min at 1600 g at 4 °C, followed by incubation at room temperature for another 30 min. After incubation, 200 µL (in duplicate) from each vial was transferred to a clear colorimetric plate. The absorbance was read at 530 nm. The values of MDA for each sample were calculated from the standard curve plotted with the corrected absorbance values. The corrected absorbance values were defined as the absorbance value of the standard A (0 µM) subtracted from itself and all other values (both standards and samples). The MDA (µM) was defined by Equation (1).

(1) $$\mathrm{MDA}\;(\text{µ}M)=\left[\frac{\left(\mathrm{corrected}\;\mathrm{absorbance}\right)-\left(y\;\mathrm{intercept}\right)}{\mathrm{Slope}}\right]$$

### 2.3. Data Collection

Baseline parameters were age, duration of subfertility (calculated in months from the moment of actively trying to conceive until the moment of intake), Body Mass Index (BMI, calculated by dividing body weight by height squared), comorbidities, medical history, medication use, educational level, smoking, illicit drug use and exposure to toxic substances (Table 1). This study divided the Dutch system’s educational level into the following 3 groups: low, average, and high. The low group includes lower education, pre-vocational secondary education, and senior general secondary education first and second year. The average group includes secondary vocational education levels 2, 3, 4, senior general secondary education and pre-university education senior years, higher professional education propaedeutic year, and university education propaedeutic year. The high group includes higher professional education, university education, and Ph.D.

In the case the specific day was unknown for the calculation of the duration of subfertility, the 15th day of the month was used. In the case males smoked loose tobacco (shag) the following was calculated: one cigarette contains around 1 g of tobacco. One bag of shag contains around 50 g of tobacco, so one bag of shag equals 50 cigarettes. One cigar contains approximately 14 g of tobacco and is therefore assumed equal to 14 cigarettes.

### 2.4. Statistical Analysis

Data were presented as means with standard deviations (SD), medians with interquartile ranges (IQR, denoted as the 1st to 3rd quartiles), or proportions *n* with corresponding percentages (%). Normality was assessed by visual inspection of histograms and normal probability (Q-Q) plots. Differences between groups were compared using independent sample *t*-tests, Mann–Whitney *U*-tests, or Kruskal–Wallis tests in the case of continuous variables, while for nominal variables chi-square tests or Fisher’s exact tests were performed, depending on normality and test assumptions. Pearson correlation coefficients were calculated to assess the correlation between two continuous variables. Subjects were subdivided into two groups of below- and above-mean seminal plasma-free thiols. Two-tailed *p*-values < 0.05 were considered statistically significant. Data analysis was performed using IBM SPSS Statistics 27.0.1.0 software package (SPSS Inc., Chicago, IL, USA). Data visualization was performed using the Python programming language (v.3.8.5, Python Software Foundation) using the *pandas* (v.1.2.3), *matplotlib* (v.3.4.1), and *seaborn* (v.0.11.1) packages in Python.

## 3. Results

### 3.1. Cohort Demographics and Characteristics

Fifty (50) males were included in this study. The demographic and clinical characteristics of the study population are presented in Table 1. The mean age of the total cohort was 35.4 (±4.9) years old. None of the participants were exposed to high-dose radiation or went to the sauna on a regular basis. In addition, there were no notable comorbidities within and exposure to toxic substances in the study population (Table S1).

### 3.2. Laboratory Results

To study potential associations between laboratory parameters and seminal plasma-free thiols, the study population was stratified by seminal plasma-free thiol level; high (above-mean) and low (below-mean). The median sperm concentration of the group with high seminal plasma-free thiol levels was significantly higher than the median of the group with low seminal plasma-free thiol levels (59.0 × 10^6^ [21.8–102.5] vs. 15.2 × 10^6^ [5.8–37.5], respectively (*p* = 0.010)) (Table 2). In addition, progressive motility was significantly higher in the high seminal plasma-free thiol group compared to the low seminal plasma-free thiol group (47.7 ± 18.4 vs. 35.2 ± 15.8, respectively (*p* = 0.017)) (Table 2). Subsequently, MDA levels were significantly higher in the high seminal plasma-free thiol group compared to the group with free thiol levels below the mean (16.9 µM [12.4–21.4] vs. 10.5 µM [5.2–15.9], *p* = 0.007) (Table 2).

### 3.3. Significant Correlations of Free Thiol and MDA Levels with Semen Parameters

**Figure 1:**
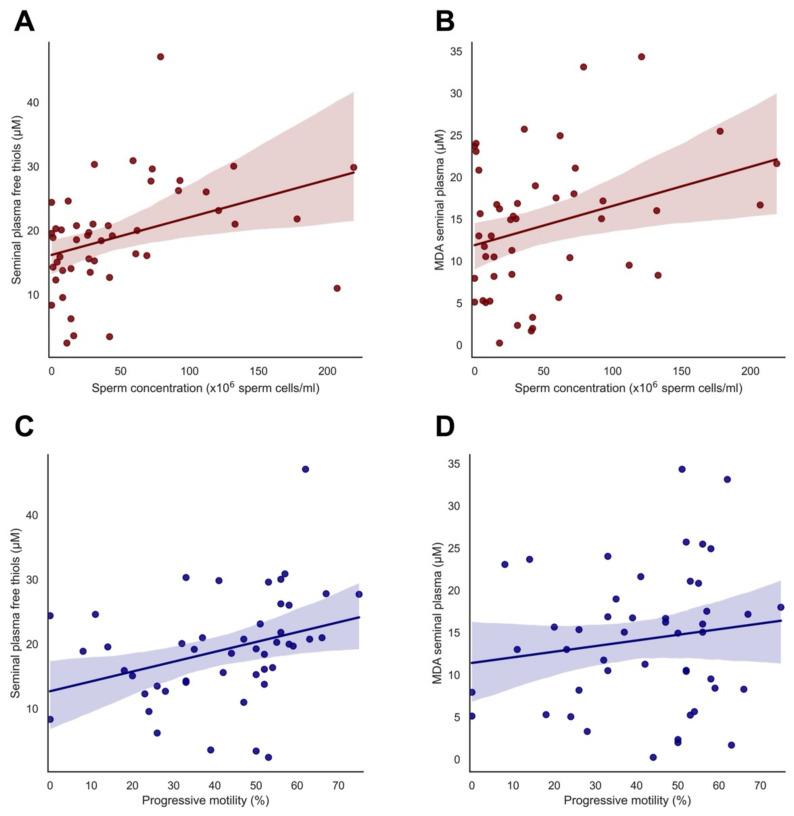
Association between seminal plasma-free thiols, seminal plasma MDA and sperm parameters: (**A**) Significant positive correlation between seminal plasma-free thiols and sperm concentration (r = 0.383, *p* = 0.008); (**B**) Significant positive correlation between seminal plasma MDA and sperm concentration (r = 0.314, *p* = 0.031); (**C**) Significant positive correlation between seminal plasma-free thiols and progressive motility (r = 0.333, *p* = 0.022); (**D**) No significant correlation between MDA seminal plasma and progressive motility (r = 0.150, *p* = 0.316). Lines represent best fitting lines from linear regression. Color-shaded areas indicate 95% confidence intervals.

A significant positive correlation was found between seminal plasma-free thiol levels and sperm concentration and progressive motility (r = 0.383, *p* = 0.008 and r = 0.333, *p* = 0.022, respectively) (Figure 1A,C). Next to this, a significant positive correlation was found between MDA levels in seminal plasma and sperm concentration (r = 0.314, *p* = 0.031) (Figure 1B), although no significant correlation was observed between MDA levels in seminal plasma and progressive motility (r = 0.150, *p* = 0.316) (Figure 1D).

### 3.4. Systemic versus Local Oxidative Stress Status

**Figure 2:**
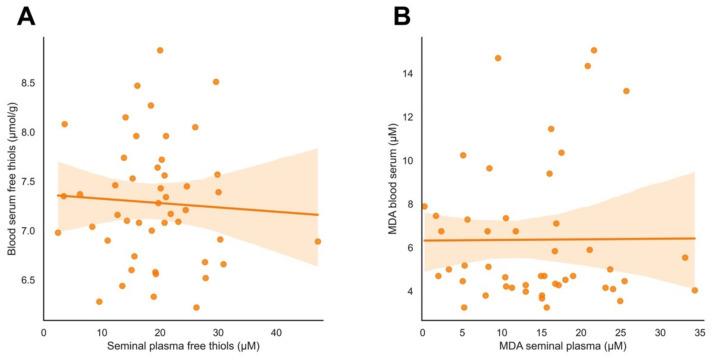
Association between systemic and local-free thiol and MDA levels. (**A**) No significant correlation between serum-free thiols and seminal plasma-free thiols (r = −0.060, *p* = 0.690); (**B**) No significant correlation between MDA levels in serum and seminal plasma (r = 0.007, *p* = 0.962). Lines represent best-fitting lines from linear regression. Color-shaded areas indicate 95% confidence intervals.

A significant positive correlation was observed between seminal plasma-free thiols and MDA in seminal plasma (r = 0.493, *p* < 0.001). However, no significant correlation was observed between serum free thiol levels and seminal plasma-free thiol levels (r = −0.060, *p* = 0.690) (Figure 2A). Likewise, no significant correlation was detected between serum MDA and seminal plasma MDA (r = 0.007, *p* = 0.962) (Figure 2B), nor between serum free thiols and serum MDA (r = 0.254, *p* = 0.075).

## 4. Discussion

In this study, we examined the association between free thiol and MDA levels in seminal plasma and semen parameters to determine whether these compounds may represent adequate biomarkers for local OS status. In addition, using these markers, we aimed to investigate whether there is an association between systemic and local OS status. First, we observed a significant positive correlation between seminal plasma-free thiol levels and sperm concentration and progressive motility. This indicates that higher free thiol levels in seminal plasma are associated with an increase in both sperm concentration and progressive motility. Furthermore, we found a significant positive correlation between MDA seminal plasma levels and sperm concentration. However, we found no correlations between the free thiol and MDA levels measured locally in seminal plasma and systemically in serum. Collectively, these findings indicate that free thiols may represent an adequate biomarker reflecting local OS status, whereas the usage of MDA as a local OS biomarker warrants further investigation. Nevertheless, local free thiol levels in the male genital tract do not seem to be associated with whole-body redox status. Although the redox interactome in the male genital tract has not yet been comprehensively characterized, the male genital tract could have its own locally controlled redox environment showing distinct differences in the levels and composition of other relevant compounds contributing to local redox status.

Our study is the first to explore free thiol and MDA levels in parallel as surrogate biomarkers for the local OS to assess semen quality (e.g., sperm concentration and progressive motility) and to compare their local and systemic levels. Our findings of local free thiol measurements and sperm concentration and motility support previous research and indicate that seminal plasma-free thiols have a positive correlation with sperm concentration and motility [18,19]. Chen et al. (2008) reported a significant increase in seminal plasma-free thiols in subfertile patients with varicocele after varicocelectomy, which positively correlated with enhanced semen quality measured as sperm motility, morphology, and density. Nonetheless, patients without improvement in semen quality also exhibited a significant increase in seminal plasma-free thiols [16]. This may suggest that an increase in antioxidant capacity is not consistently associated with an increase in semen parameters, such as sperm motility and concentration. Following this, Shiva et al. (2011) reported a positive correlation between free thiol levels and increasing ranges of sperm count. However, they found no significant correlation between free thiols in seminal plasma and sperm motility and morphology [19]. Thus, existing literature inconsistently reports on the correlation between free thiol levels and sperm motility, concentration, and morphology [17,30].

Furthermore, we found a significant positive correlation between MDA seminal plasma levels and sperm concentration, which also aligns with findings in existing knowledge [22,23]. Collodel et al. (2014) found a positive correlation between sperm concentration and MDA levels and a negative correlation between sperm motility and MDA levels, whereas Micheli et al. (2019) found a positive correlation between both sperm concentration and motility and MDA concentration. However, MDA is a byproduct of lipid peroxidation, and therefore higher MDA levels should be expected to indicate the presence of more OS and, consequently, results in unfavorable semen parameters [10]. Multiple hypotheses could explain the positive correlation between MDA and sperm concentration found in our study. Firstly, higher concentrations of spermatozoa suggest more ROS-producing sources, initiating a higher level of lipid peroxidation and therefore an increase in MDA levels [31]. Secondly, in our study, the MDA concentration is significantly higher in the group with high seminal plasma-free thiol levels compared to low seminal plasma-free thiol levels (Table 2). In line, there was also a significant positive correlation between seminal plasma-free thiol and MDA levels. It could be contemplated that as a reaction to increased lipid peroxidation of the cell membranes of spermatozoa, more free thiols are produced and/or released as a compensatory response via unknown mechanisms. This hypothesis could explain the correlation we found between MDA levels and sperm concentration. However, since the present study was of cross-sectional origin, no cause-and-effect relationships could be revealed that may better explain these findings. Further research is warranted to unravel the exact regulatory mechanisms by establishing causality and to shed light on the relative contributions of each individual redox-related compound to sperm quality.

No association was found between blood serum-free thiol and MDA levels and seminal plasma-free thiol and MDA levels (Figure 2). This may indicate that systemic OS and local OS are not strongly interrelated and support the notion that the male genital tract may have a local redox-controlled microenvironment that is responsible for the overall redox balance. It is noteworthy that matured spermatozoa show different behavior from other body cells because of their function in fertilization, making them more susceptible to oxidative stress than other systemic cells. Examples of divergent mechanisms are the spermatozoa’s limited antioxidant protection and production due to the limited volume of the cytoplasm. During maturation, spermatozoa expel excess cytoplasm as a residual body to decrease load and, therefore, boost motility. Consequently, they are dependent on the presence of antioxidants within seminal plasma produced by the secretory glands. For the same reason, spermatozoa inactivate many repair mechanisms, for example, for DNA, and therefore are more vulnerable to oxidative DNA damage. These matters are specific to germ cells locally and can possibly explain why there is no correlation between systemic and local OS [32]. The question that arises is how the systemic state, such as proven lifestyle factors such as excessive alcohol use and smoking, influences male infertility [33,34]. Excessive alcohol consumption is reported to increase OS within the testis as a result of a significant reduction in testosterone, an increase in serum lipid peroxidation byproducts, and a decrease in antioxidants [35]. However, no study to date has directly examined the link between several lifestyle and environmental factors and local oxidative damage.

Strengths of our study include that we evaluated the combination of two commonly used biomarkers for OS and compared them systemically and locally in the male genital tract. Correlating free thiol and MDA levels and measuring them systemically and locally is unique for this study and provides information on the reliability of free thiols and MDA as biomarkers for the local OS. Usage of a biomarker for OS can be of importance to establishing possible explanations for idiopathic male infertility. Another strength of our study is that these two biomarkers represent different components of the RSI. Free thiols constitute the central hubs of the extracellular antioxidant machinery, reflecting the whole-body redox state, whereas MDA is merely a damage marker representing OS-induced lipid peroxidation. Measuring the combination of free thiols and MDA gives a more accurate representation of the overall redox state within seminal plasma and serum. Even though many different OS biomarkers are currently in use, further research is necessary to define their relative contributions, their purposes, and their interactions within the different levels of the redox interactome [36].

There were also some limitations to this study that warranted recognition. Firstly, the small sample size could lead to ambivalent results. A larger sample size should also consist of males from the general population, i.e., males that are not trying to conceive. They might be less conscious of their lifestyle (having perhaps a high BMI or smoking heavily) and therefore have a possibly higher systemic OS than those present in this study population. Broadening and enlarging the study population could still expose a relation between systemic and local OS. Secondly, for the seminal plasma-free thiols, the absolute values were displayed. These values were not corrected for protein content, as is usually performed for systemic free thiol measurements. Regarding blood serum-free thiol levels, these concentrations are corrected for albumin as this is the most dominant plasma protein, determining the quantity of detectable free thiols [28]. However, the composition and dynamics of the seminal thiol pool are not yet fully uncovered, which complicates the rationale for adjusting for protein or albumin content. Nevertheless, the results of this study provide an important stepping stone for more research in the field of OS related to male infertility.

Furthermore, based on the knowledge of existing OS, interventions and therapies could be applied to lower local OS. Seminal plasma-free thiols can then serve as a surrogate measure for the beneficial effects of these interventions, thereby monitoring the reduction of the local OS.

Currently, many aspects are still unknown about the exact protective mechanisms of the male genital tract against oxidative stress. In this study, we have shown that free thiols in seminal plasma might be a reliable marker to reflect local redox status. Future research could include reproductive hormones to relate the hormones to local redox status and semen parameters such as sperm concentration and motility. In addition, in order to achieve a more complete overview of the redox status, additional markers of oxidative stress could be measured, including stable end-products of ROS (e.g., peroxides, lipid peroxidation products, protein carbonyl species, or oxidized DNA products), reactive nitrogen species (RNS, e.g., nitrite and nitrate) and reactive sulfur species (RSS, e.g., thiosulfate and sulfate).

Furthermore, knowledge about the origin of antioxidants locally would provide more insight into the defense system of the local OS. An implication for future studies is to examine free thiols in fractions of prostate fluid and fluid from seminal glands and compare them with the samples from the end-product of seminal plasma. This might indicate where the free thiols are mainly produced. Furthermore, the cause-and-effect mechanism behind OS-induced processes needs extra research. This gives us more information about the relation between sperm parameters, such as sperm motility, concentration, and morphology, and the measured OS biomarkers. Finally, many studies state that several lifestyle and environmental factors have a negative influence on sperm quality, e.g., sperm concentration, motility, and morphology. However, further research needs to focus on establishing the potential role of OS in the links between environmental factors and sperm quality.

## 5. Conclusions

This exploratory study demonstrates that seminal plasma-free thiols constitute a promising biomarker, reflecting local OS associated with sperm concentration and motility. The relevance of MDA as a biomarker for OS in seminal plasma needs further research. Furthermore, this study observed no relation between systemic and local OS status. However, future studies are warranted to clarify the exact composition of the redox interactome of the male genital tract and define the relative contributions of relevant redox compounds in shaping local OS status.

## Figures and Tables

**Table 1:** Demographic and clinical characteristics of the study population.

	Total *n* = 50
**Age** (years)	35.4 ± 4.9
Duration of subfertility (months)	15 [12.0–19.0]
BMI (kg/m^2^)	25.2 ± 4.2
**BMI groups**	
Underweight (BMI < 20)	3 (6)
Healthy (BMI 20–25)	25 (50)
Overweight (BMI 25–30)	16 (32)
Obese (BMI ≥ 30)	6 (12)
**Educational level**	
Low	4 (8)
Average	14 (28)
High	32 (64)
**Medication use**	
Type of medication ^(1)^	10 (20)
**Type of subfertility**	
Primary	35 (70)
Secondary	15 (30)
**SA type** ^(2)^	
Normozoospermia	30 (60)
Oligozoospermia	6 (12)
Asthenozoospermia	2 (4)
Oligoasthenozoospermia	12 (24)
**Smoking**	
Never	23 (46)
Past	
1–9 cigarettes/day	7 (14)
≥10 cigarettes/day	10 (20)
Current	
1–9 cigarettes/day	4 (8)
≥10 cigarettes/day	8 (16)
Smoking years of past and present users	10.0 [2.5–15.5]
**Alcohol use**	40 (76.9)
Units per week	3.3 [2.0–7.5]
**Drug use**	
Never	30 (60)
**Soft drugs**	
Past	19 (38)
Current	5 (10)
**Hard drugs**	
Past	8 (16)
Current	0
Exposure to toxic substances at work ^(3)^	13 (74)
Experience stress at work	4 (8)
Warm baths 1 time per week	6 (12)
Mean testicular volume (mL) ^(4)^	20.6 (11.2)

Data are presented in mean ± SD, median [p25–p75], or *n* (%). ^(1)^ The types of medication included the following: budesonide, dovobet (calcipotriol/betamethasone), betamethasone, omeprazole, and antidepressant. For the above mean group, the types of medication included budesonide, Lexapro (escitalopram), salbutamol, elocon, cetirizine, triamcinolone, vit. D, vit. D&C. ^(2)^ Classification is defined by the WHO guidelines [23]. ^(3)^ Exposure to toxic substances present in the study population are stated in Table S1. ^(4)^ For mean testicular volume, values were missing due to inconsistent registration at the intake, see Table S2.

**Table 2:** Laboratory results of the study population.

	Total *n* = 50	Low Seminal Plasma-Free Thiol Levels *n* = 22	High Seminal Plasma-Free Thiol Levels *n =* 25	*p*-Value
Seminal plasma-free thiols ^(1)^ (µM)	19.1 ± 8.4	12.5 ± 4.9	24.9 ± 6.2	<0.001 *
Blood serum-free thiols (µmol/g)	7.3 ± 0.6	7.3 ± 0.6	7.3 ± 0.6	0.923
MDA seminal plasma ^(1)^ (µM)	15.1 [8.2–19.0]	10.5 [5.2–15.9]	16.9 [12.4–21.4]	0.007 *
MDA blood serum (µM)	5.0 [4.2–7.4]	4.7 [4.1–6.8]	5.5 [4.4–9.9]	0.090
Period of abstinence (days)	3.1 ± 1.3	3.4 ± 1.5	3.0 ± 1.0	0.334
Sperm concentration (×10^6^ sperm cells/mL)	29.2 [7.4–72.3]	15.2 [5.8–37.5]	59.0 [21.8–102.5]	0.010 *
Progressive motility (%)	40.9 ± 18.4	35.2 ± 15.8	47.7 ± 18.4	0.017 *
Consistency of semen				0.722
Moderately mucous	47 (94)	21 (95.5)	24 (96)	
Highly mucous	3 (6)	1 (4.5)	1 (4)	
pH ^(1)^	7.6 ± 0.2	7.6 ± 0.1	7.6 ± 0.2	0.750
Round cells ^(1)^	0.5 [0.2–1.0]	0.6 [0.2–1.3]	0.4 [0.2–1.0]	0.666
Total semen volume (mL)	3.3 ± 1.6	3.3 ± 1.6	3.6 ± 1.5	0.587
Albumin concentration in blood serum (g/L)	47.0 [45.8–48.0]	47.0 [45.0–48.0]	47.0 [46.0–48.0]	0.559
Albumin concentration in seminal plasma ^(1)^ (g/L)	0.7 [0.6–0.9]	0.7 [0.6–0.8]	0.7 [0.5–0.9]	0.779

The values are presented in mean ± SD, median [p25–p75] or *n* (%) stratified by the total cohort, low seminal plasma-free thiol levels, and high seminal plasma-free thiol levels. ^(1)^ For these parameters, values were missing due to measurement deficiencies (see Table S2). * Two-tailed statistically significant with *p* < 0.05.

## Data Availability

The data presented in this study are available on request from the corresponding author. The data are not publicly available due to privacy and ethical considerations.

## Supplementary Materials

The following supporting information can be downloaded at: https://www.mdpi.com/article/10.3390/antiox11061045/s1, Table S1: Exposure to toxic substances at work and comorbidities in the present and/or past among the study population. Table S2: Missing values of clinical characteristics and laboratory analysis.
