# Supplementary material for: Local and Systemic Oxidative Stress Biomarkers for Male Infertility: The ORION Study
Source: Antioxidants (Basel). 2022 May 25;11(6):1045. doi: 10.3390/antiox11061045 (PMC9220279; doi:10.3390/antiox11061045)
Supplement: Supplementary file 1 [file antioxidants-11-01045-s001.zip › antioxidants-1721725-supplementary-done.pdf]

antioxidants

Table S1. Exposure to toxic substances at work and comorbidities in the present and/or past among the study population.

Exposure to Toxic Substances	Total *n* = 50	Comorbidities	Total *n* = 50
High temperature	4 (8)	Stomach ulcer	1 (2)
Oils/Tar	3 (6)	Thyroid condition	1 (2)
Toxic solvents	4 (8)	Kidney condition	1 (2)
Toxic damps	7 (14)	Psychiatric complaints	3 (6)
Vinylchloride	1 (2)	Inflammatory bowel syndrome	1 (2)
Arsenic	1 (2)	Allergy (incl. hay fever)	5 (10)
Heavy metals	2 (4)	Asthma	2 (4)
Lead	2 (4)	Eczema/psoriasis	3 (6)
Cadmium	1 (2)		
Mercury	1 (2)		
Dyes/pigment	1 (2)		
Pesticides	4 (8)		
Herbicides	3 (6)		
Fungicides	2 (4)		
Cyanides	1 (4)		
Plastics	2 (4)		

Data is presented in *n* (%).

Table S2. Missing values of clinical characteristics and laboratory analysis.

	Total *n* = 50
Mean testicular volume	13 (26)
Seminal plasma free thiols	3 (6)
pH	2 (4)
Round cells	10 (20)
Albumin concentration in seminal plasma	4 (8)
MDA seminal plasma	3 (6)

Data is presented in *n* (%).
